# Electroless deposition of tellurium nanowires in eutectic solvents using immobilised silver islands†

**DOI:** 10.1039/d2ra06356e

**Published:** 2022-12-15

**Authors:** Samuel C. Perry, Joshua White, Iris Nandhakumar

**Affiliations:** Department of Chemistry, University of Southampton Southampton UK iris@soton.ac.uk

## Abstract

In this work we demonstrate a new approach towards the electroless deposition of tellurium nanowires in deep eutectic solvents. Unlike most electroless deposition where the substrate is sacrificed to drive the reduction, our process uses immobilised silver epoxy islands on gold films to give localised galvanic displacement of the silver, resulting in an even growth of wires across the entire gold electrode surface. We demonstrate the strong dependence of the nanostructure on the experimental conditions, with changes in bath temperature, tellurium concentration and the halide component of the solvent leading to sizeable alterations in the nanowire geometry. This demonstrates electroless deposition as a promising synthetic route towards low-dimensional tellurium nanostructures.

## Abstract 2

In this work we demonstrate a new approach towards the electroless deposition of tellurium nanowires in deep eutectic solvents.

## Graphical abstract

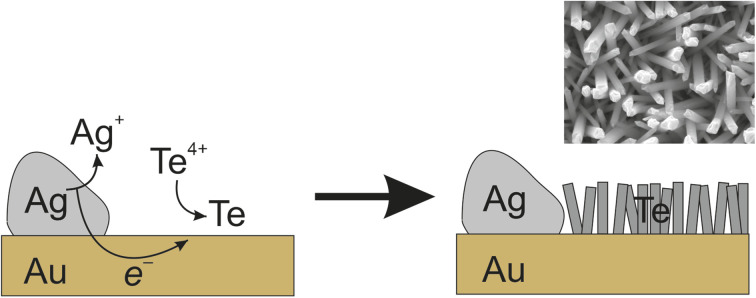

## Introduction

Tellurium (Te) nanostructures are leading materials in a number of fields in a broad range of applications thanks to their interesting thermoelectric, piezoelectric and optoelectronic properties.^1,2^ Te nanostructures outperform analogous bulk materials, leading to a sizeable interest in developing new means of producing low-dimensional materials.^3,4^

Nanowires are commonly made *via* solvothermal synthesis, which give low order structures at good yields, but also requires complex and costly high temperature and vacuum conditions to achieve a reasonable efficiency.^5^ Electrodeposition through a porous template can produce ordered arrays of similar wires, though challenges of pore filling can hinder growth, particularly for depositions into pores on the order of tens of nanometers.^6^

Template-free electrodeposition is a promising alternative that can produce small diameter nanowires without complex templates, high temperatures or vacuum apparatus. Reaction conditions are chosen to guide growth favoured crystal direction by dissolved species such as SiCl_4_ (ref. 7) or halides.^8,9^ Unfortunately, this requirement leads to examples of template-free Te nanowire fabrication being limited to costly ionic liquids.

In this work, we use deep eutectic solvents (DES) as a cost effective alternative to produce arrays of tellurium nanowires. DES offer the same advantages as ionic liquids (high solubility, wide solvent window and high conductivity) but at a reduced cost.^10^ Only a handful of works have investigated Te electrodeposition in DES,^11,12^ which were able to produce interesting nanostructures but were unable to extend this to significant wire growth.

We addressed this challenge by producing Te nanowire films using electroless deposition, where galvanic displacement facilitates Te nanowire growth. In this process, a solid metal is immersed in a solution of a precursor cation, where the precursor has a more positive standard potential compared to the solid. The difference in standard potential provides a thermodynamic driving force that causes the solid metal to oxidise and dissolve and the precursor cation to reduce and deposit.

**Fig. 1:**
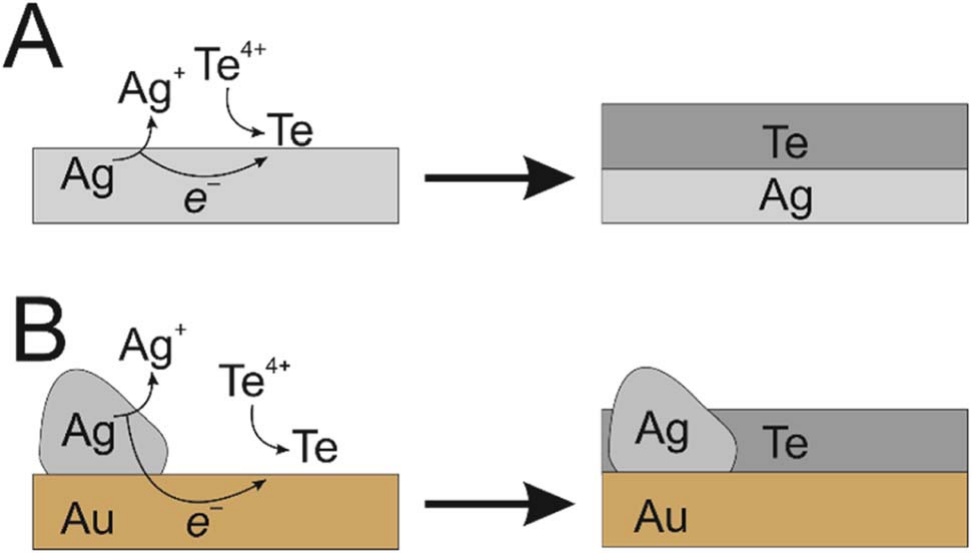
Schematic electroless deposition. (A) The commonly employed method – a sacrificial substrate undergoes galvanic displacement. (B) The new proposed method where immobilised Ag islands on an inert Au substrate drives the same reaction. Charge balance is not represented for simplicity.

Traditionally, it is the substrate itself that dissolves to facilitate the electroless deposition on that same surface.^13^ We have taken a novel approach to achieve this same reaction with immobilised silver epoxy on the surface of a gold electrode. The high conductivity of both the silver epoxy and gold layer allowed galvanic displacement of the silver to drive the formation of Te nanowires across the entirety of the gold surface, producing an even coating (Fig. 1). This is analogous to the use of sacrificial zinc on steel components for marine applications, where zinc provides galvanic protection against steel corrosion, since the zinc will preferentially oxidise.^14^ However, in our case the bolted-on sacrificial material is being used to drive an electrodeposition. Although galvanic deposition of Te in general is well known, to the best of our knowledge, this is the first time that electroless deposition has been achieved on an inert substrate in this way, where electrically connected sacrificial material drives electrodeposition on an inert conductive substrate.

We present the impact of Te concentration, temperature and produce bromide and iodide analogues of a popular chloride-based DES to show the impact of halides on wire growth. Although some works have investigated the impact of low concentrations of different halides by changing the tellurium halide precursor,^8,9^ we believe this is the first example where DES is produced entirely with the alternative halide salt to show the impact of larger concentrations of the halide in question. We propose DES electroless deposition is a facile route to high quality Te nanowire arrays.

## Experimental

### Materials

All solutions were weighed and prepared in a nitrogen flow box (Cleaver Scientific). Deep eutectic solvents (DES) were produced by stirring 1 mol% choline chloride (99%, Acros Organic) with 2 mol% ethylene glycol (99%, Fisher) at 60 °C until fully dissolved. The choline chloride-based eutectic was designated 12CE-Cl.^15^ Bromide and iodide analogues were made using the same procedure with choline bromide (98%, TCI) and choline iodide (98% Alfa Aesar) and were designated 12CE-Br and 12CE-I respectively. Working solutions were produced by dissolving either TeCl_4_ (99%, Aldrich), TeBr_4_ (99.9%, Alfa Aesar) or TeI_4_ (99%, Alfa Aesar) in the DES with the matching halide at 60 °C with magnetic stirring.

### Electrode fabrication

Gold film electrodes were produced in house. Glass slides were first cleaned by sonicating for 30 min sequentially in Decon® detergent, deionised water and isopropyl alcohol and then dried under nitrogen. A 20 nm chromium layer was first sputtered in order to aid the adhesion between glass and gold, before a 100 nm gold layer was sputtered on top. The glass slides were cut into 1 cm × 1.5 cm pieces with a glass scribe. Electrodes for the deposition were prepared by immobilising silver in the form of silver epoxy (RS components) as a thin strip onto the edge of surface of the gold film electrode.

### Electroless deposition

Electroless deposition was achieved by first warming the DES to the desired temperature in a sealed glass vial immersed in an oil bath. Once the desired temperature was reached, the electrode was gently lowered into the vial and resealed. A dark grey deposit was seen to rapidly form, which became progressively thicker over time. Once the required length of time had passed, the electrode was removed and sequentially washed in deionised water and isopropyl alcohol and then dried with nitrogen.

Images and elemental analysis of the nanowire films were recorded on a Jeol JSM-7200F scanning electron microscope (SEM) equipped with energy dispersive X-ray (EDX) capability. The crystalline structure was investigated by Xray diffraction (XRD) using a Bruker D2 Phaser benchtop diffractometer (300 W Cu sealed tube) at 45 kV and 150 mA.

## Results and discussion

### Electroless deposition from chloride-based DES

**Fig. 2:**
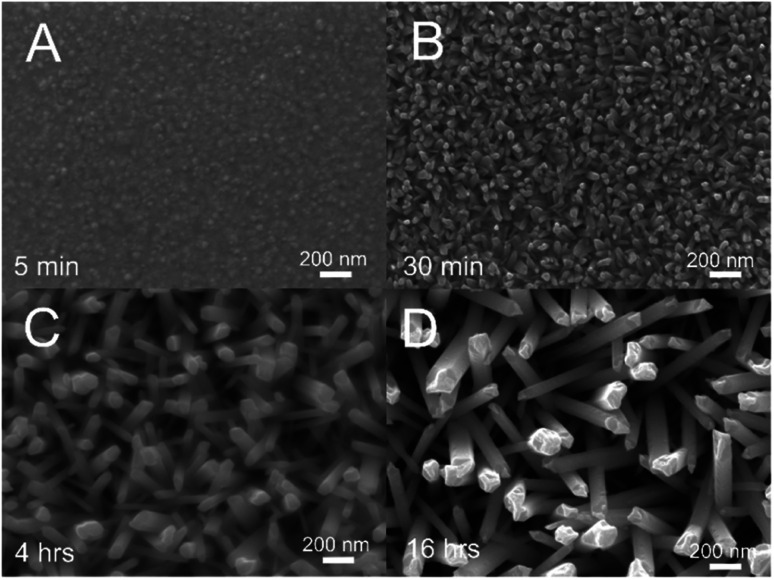
Te nanowires from electroless deposition on a gold electrode in 5 mM TeCl_4_ in 12CE-Cl at 80 °C. Deposition was for 5 min (A), 30 min (B) 4 h (C) and 16 h (D).

Preliminary investigations into the Te nanowires growth were carried out in 5 mM TeCl_4_ DES made up of 1 : 2 choline chloride : ethylene glycol (12CE-Cl) at 80 °C (Fig. 2). Electrodes were removed from the deposition bath after 5 minutes, 30 minutes and 4 hours in order to demonstrate the phases of wire growth.

After only 5 minutes deposition time (Fig. 2A) a thin film was produced across the entire surface of the electrode featuring the nucleation sites for further growth into nanowires. The progress can be seen after 30 minutes (Fig. 2B) where short nanorod structures are seen on the electrode, which continue to grow into wires after 4 (Fig. 2C) and 16 hours of immersion (Fig. 2D). Diameters of the rods at 30 min were in the range of 20 to 40 nm, which increased as the wires grew in length to 30 to 70 nm after 4 hours and 50 to 90 nm after 16 hours. The aspect ratio (wire length/diameter) was between 20 and 30 for the observed nanostructures. As a brief note, the distinction between nanorods and nanowires has been defined as wires having an aspect ratio greater than 20,^16^ so these nanostructures could be defined as long rods or short wires, though there is potential for continued growth here by extending the deposition time.

The slight increase in thickness with increased deposition time is likely highlighting the level of effectiveness of the 12CE-Cl DES in guiding the formation of nanowires as opposed to other structures. Longer deposition times give a slight increase in wire thickness but a large increase in wire length, suggesting the DES can favourably produce wires but the coordination effect does not prevent all lateral growth.

Cross sectional imaging across a 200 μm section of the 4 hour sample revealed an average film thickness of approximately 1.3 μm corresponding to a growth rate of 325 nm h^−1^, although the actual rate in terms of wire length is faster since the wires are slanted and interwoven. Elemental analysis using energy dispersive X-ray spectroscopy (EDX, ESI Fig. S1†) showed strong signals for tellurium, but no signal for silver, which gives confidence that galvanically displaced silver from the epoxy does not contaminate resultant nanostructures. Further crystallographic analysis (ESI Fig. S4†) confirms the production of a pure Te phase, with sharp peaks suggesting a high degree of crystallinity.

### Influence of concentration and temperature

**Fig. 3:**
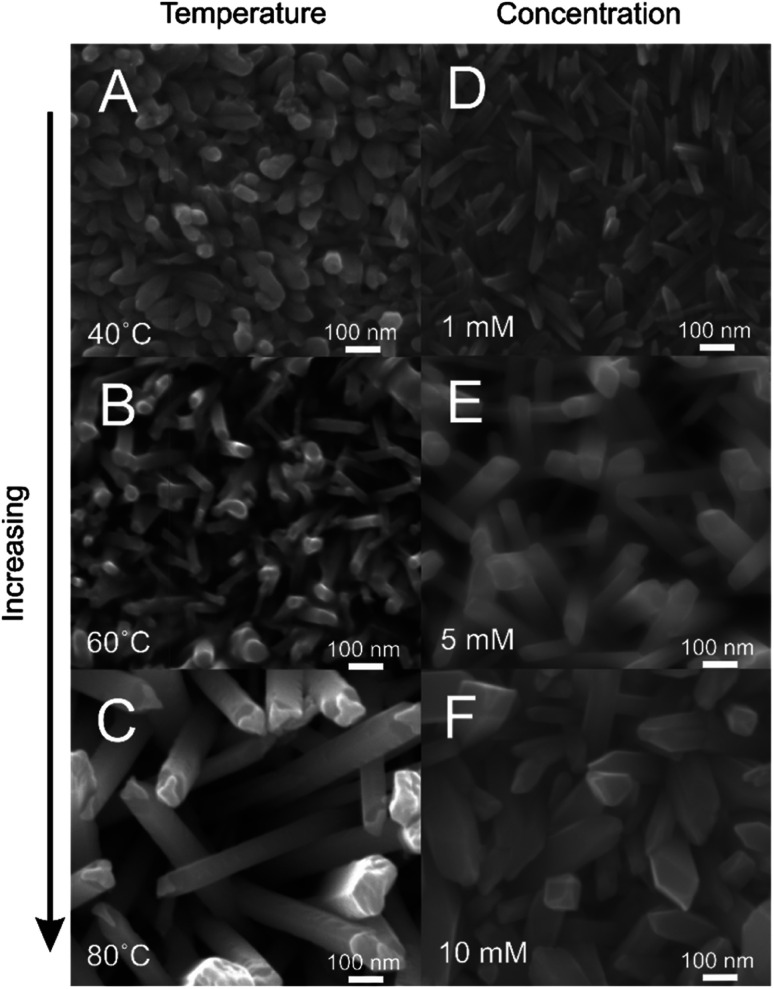
Tellurium nanowires formed *via* electroless deposition on a gold film electrode in TeCl_4_ in 12CE-Cl where key parameters were modified. (A–C) Deposition was done in 5 mM TeCl_4_ at 40 °C (A) 60 °C (B) and 80 °C (C) for 16 hours. (D–F) Deposition was done at 80 °C with 1 mM (D), 5 mM (E) and 10 mM (F) TeCl_4_ for 4 hours.

Further parameterisation of the electroless deposition procedure revealed a strong dependence of the obtainable nanostructure on the precise conditions used. Electroless Te nanowire growth in identical 12CE-Cl solutions at 40, 60 and 80 °C (Fig. 3A–C) shows that in increase in bath temperature lead to an increase in growth rate, most likely due to the enhanced mass transfer rate of Te^4+^ to the gold surface. The lower temperature deposits also showed features with smaller diameters in the range of 20 to 40 nm at 40 °C *vs.* 50 to 90 nm at 80 °C. The lower temperature deposits also showed a greater degree of fusion between neighbouring wires, whereas the 80 °C sample showed more distinct and ordered wires, suggesting that the higher mass transport rate is needed for wire growth.

Similarly, wire growth was strongly dependent on concentration (Fig. 3D–F). Lower concentrations led to features that were thin and needle like with diameters 20 to 30 nm, whereas higher concentrations produced much larger features that more resembled platelets than wires, with a broad variance in geometries well in excess of 100 nm. The lower concentration also produced wires that were more likely to be fused as opposed to freestanding. These trends are similar to those produced by varying the bath temperature, which agrees with the rate of Te^4+^ transport being key in determining the nature of the nanostructure formed by electroless deposition.

### Electroless deposition from bromide and iodide-based DES

**Fig. 4:**
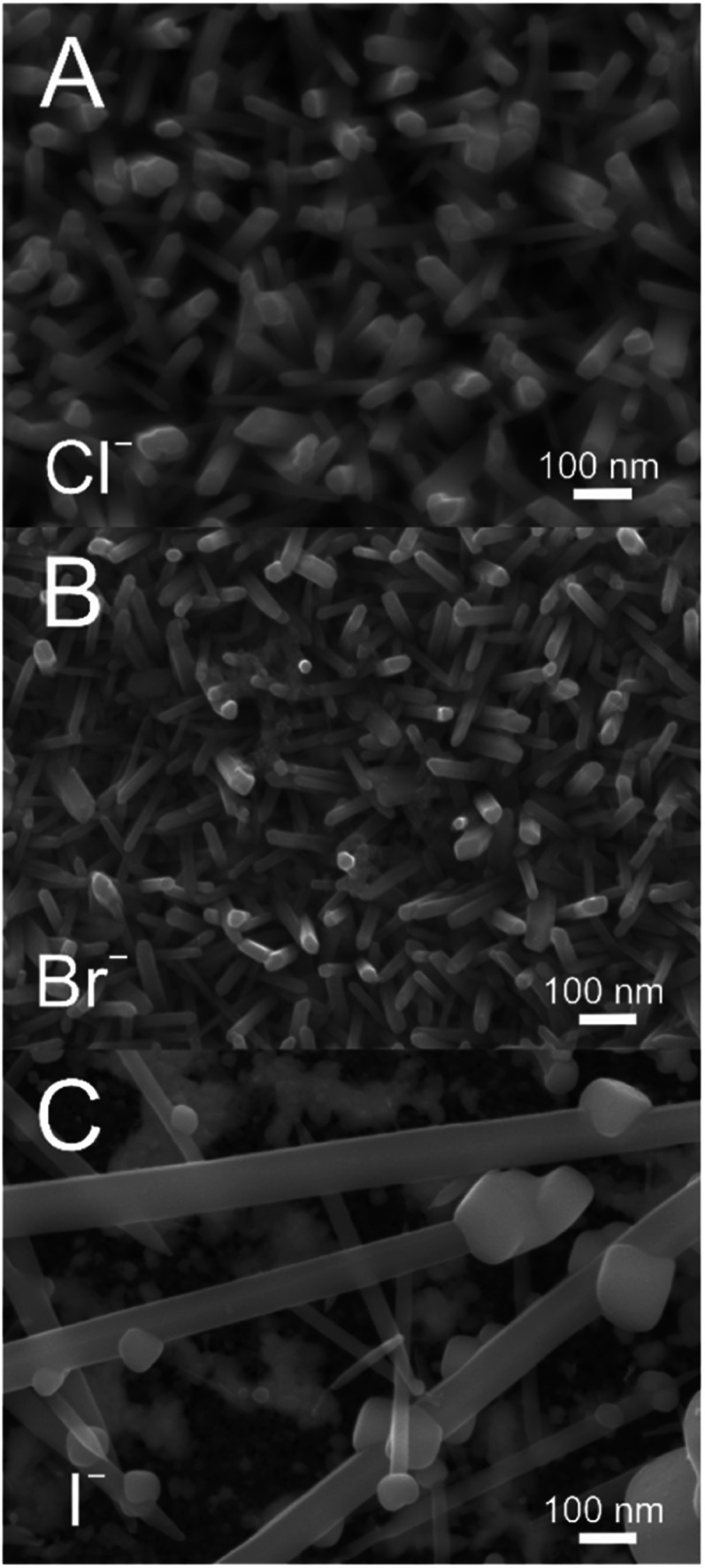
Tellurium nanowires formed *via* electroless deposition on a gold film electrode in 5 mM TeX_4_ in 12CE-X at 80 °C where the halide ‘X’ was varied. Deposition was for 4 hours with TeCl_4_ (A), TeBr_4_ (B) and TeI_4_ (C).

Analogous DES to 12CE-Cl using choline bromide (12CE-Br) and iodide (12CE-I) were used to dissolve TeBr_4_ and TeI_4_ respectively for electroless deposition (Fig. 4). The nanowire growth is seen to be strongly impacted by the choice of halide in this case. Nanowires grown in 12CE-Cl and 12CE-Br are comparable in terms of size and length, although the 12CE-Br bath produced wires with slightly smaller geometries in the 25 to 40 nm range compared to 30 to 70 nm in 12CE-Cl. 12CE-I, on the other hand, lead to a highly erratic deposition, with a few extremely large wires dispersed over the electrode surface. The adhesion of films grown in 12CE-I was also significantly poorer than in both the 12CE-Cl and 12CE-Br cases, resulting in a large amount of tellurium mass loss when the sample was removed from the deposition bath.

The difference in deposition when varying the halide also provides insights into the nature of the galvanic displacement reaction. The driving force for electroless deposition is the difference in standard potentials of the immobilised Ag *versus* the dissolved Te.^17^ In order for Te deposition to be spontaneous, the standard potential (*E*^0^) for the Ag couple must be more negative than for the Te.Te^4+^ + 4e^−^ ⇌ Te^0^, *E*^0^ = 0.568 V *vs.* RHEAg^+^ + e^−^ ⇌ Ag^0^, *E*^0^ = 0.800 V *vs.* RHEAgCl + e^−^ ⇌ Ag^0^ + Cl^−^, *E*^0^ = 0.222 V *vs.* RHEAgBr + e^−^ ⇌ Ag^0^ + Br^−^, *E*^0^ = 0.071 V *vs.* RHEAgI + e^−^ ⇌ Ag^0^ + I^−^, *E*^0^ = −0.152 V *vs.* RHE

Based on this, the appropriate Ag component must be the halide, not Ag^+^, since only the reaction with AgCl would be spontaneous. We can also use this to rationalise the main differences between Te deposition in Cl^−^, Br^−^ and I^−^-based DES. Moving to the larger halides makes *E*^0^ for the Ag couple progressively more negative, resulting in a larger thermodynamic driving force for the electroless deposition; *E*^0^ of the reaction increases from 346 mV in Cl^−^ to 497 mV and 720 mV in Br^−^ and I^−^, respectively. The difference between Cl^−^ and Br^−^ is relatively minor, suggesting that there is a reasonably wide potential window where nanowires can be formed *via* electroless deposition. However, moving to I^−^ makes the driving force larger enough to completely change the nature of the deposit, resulting in a few extremely large wires dispersed across the electrode surface. This has implications for the future electroless deposition of alternative materials by the same methods, with a preliminary upper limit of 500 mV for *E*^0^ of the overall reaction.

Interestingly, the production of smaller geometry wires in Br^−^ seems to be at odds with the observed impact of concentration and temperature. Increasing concentration or temperature to increase the mass transport of Te^4+^ led to features with larger diameters, whereas the increased driving force due to the AgBr/Ag + Br^−^ redox couple appears to produce smaller diameters. This may indicate a further role of the halides on nanowire growth as capping agents to help direct growth along the wire rather than growing outwards and broader.^18^ This would make the bromide analogue of the more commonly employed choline chloride DES a promising target for producing low diameter nanowires.

This could potentially be employed to nanowires of other strategically interesting metals by replacing the tellurium halide with the corresponding metal salt. It is worth mentioning that a fair amount of optimisation is needed in terms of deposition bath conditions and precursor concentrations, and so moving to an alternative metal is not facile. However, with due consideration to the deposition bath conditions and by selecting an appropriate redox couple based on the standard potentials, we believe this technique could be expanded to a broader range of materials. To date, we have demonstrate this only with commercial silver epoxy, but the immobilisation of alternative sacrificial metals could also open this technique to an even wider range of materials by offering a broader scope of electrode potentials.

## Conclusions

In summary, we have demonstrated the electroless deposition of tellurium nanowires using in DES. For the first time, we have used silver epoxy on gold electrodes as a simple means of immobilising silver for galvanic displacement, allowing electroless deposition at a noble gold surface. Using a 12CE-Cl as a standard DES, we demonstrated that taking steps to decrease the availability of Te^4+^ to the electrode surface, such as through decreased concentration of temperature, leads to smaller geometries in the resultant nanostructures, although this also allows neighbouring nanostructures to become fused leading to a more complex film structure.

We have also showed that halide analogues of 12CE-Cl employing Br^−^ and I^−^ choline salts result in vastly different nanostructures, with Br^−^ reducing the wire diameter but I^−^ massively increasing it. We believe this relationship is due to the complex balance between two factors. A difference in the standard potentials for the AgBr and AgI redox couples leads to a greater driving force for deposition in 12CE-I *vs.* 12CE-Br. This alone would give a diameter trend where Cl^−^ < Br^−^ < I^−^ but the observed trend is actually Br^−^ < Cl^−^ < I^−^, suggesting that Br^−^ is able to act as a capping agent to drive the formation of small diameter nanowires and counter this impact. This presents DES, particularly those with high concentrations of Br^−^, as promising solvents for template-free production of metallic nanowires by electroless, and potentially also by electrochemical methods.

## Conflicts of interest

There are no conflicts to declare.

## Supplementary Material

RA-012-D2RA06356E-s001
