# Supplementary material for: Electroless deposition of tellurium nanowires in eutectic solvents using immobilised silver islands
Source: RSC Adv. 2022 Dec 15;12(55):35938–42. doi: 10.1039/d2ra06356e (PMC9752427; doi:10.1039/d2ra06356e)
Supplement: RA-012-D2RA06356E-s001 [file RA-012-D2RA06356E-s001.pdf]

Electronic Supplementary Material (ESI) for RSC Advances.
This journal is © The Royal Society of Chemistry 2022

Electronic Supplementary Information

Electroless deposition of tellurium nanowires in eutectic solvents using immobilised silver islands

Samuel C. Perry, Joshua White and Iris Nandhakumar

Department of Chemistry, University of Southampton, Southampton, UK.

Energy Dispersive X-Ray (EDX) Spectroscopy

Elemental analysis of the tellurium films was done for electroless depositions from deep eutectic solvents (DES). All analysed films were produced from a solution of 5 mM tellurium halide dissolved in a DES made up of 1 mol% choline halide with 2 mol% ethylene glycol. These samples are designated 12CE-X, where X is wither Cl, Br or I depending on the halide used. Depositions were done in a thermostatically controlled bath at 80°C for 4 hours. In all cases, no silver was observed confirming that silver from the immobilised epoxy did not contaminate the deposited tellurium. The oxygen signal indicates that the analysed tellurium films were oxidised, which likely occurred during the transport of the film from fabrication to analysis, which was done under ambient conditions. The tellurium signal is significantly decreased in the case of iodide due to challenges with delamination and loss of material in 12CE-I that was not present with 12CE-Cl or 12CE-Br.

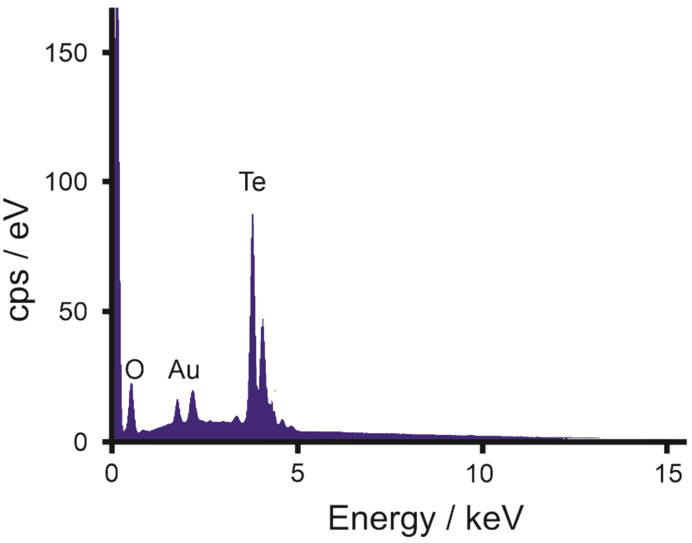

Figure S1: EDX spectra recorded for a film of tellurium nanowires produced via electroless deposition from 5 mM $TeCl_4$ in 12CE-Cl DES at 80°C.

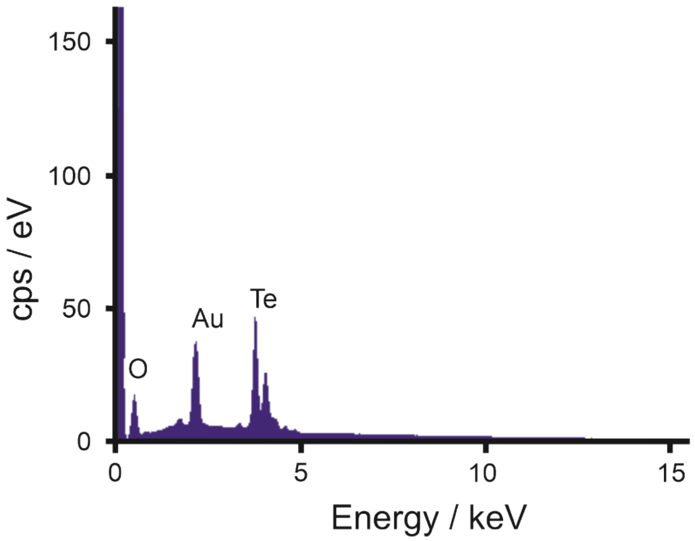

Figure S2: EDX spectra recorded for a film of tellurium nanowires produced via electroless deposition from 5 mM $TeBr_4$ in 12CE-Br DES at 80°C.

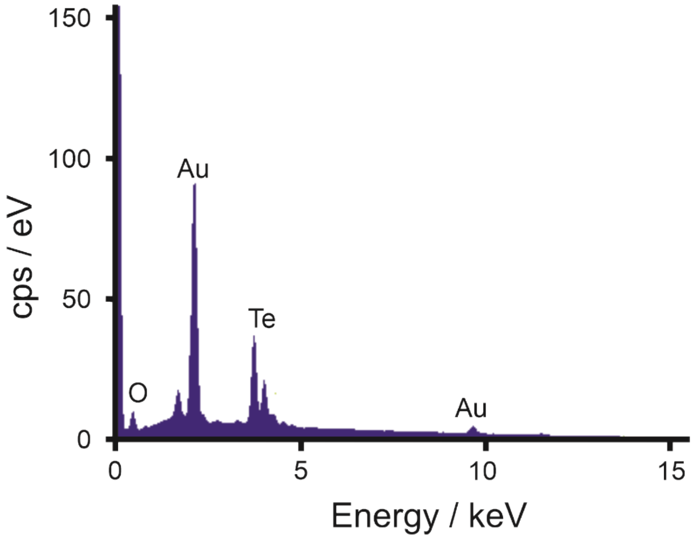

Figure S3: EDX spectra recorded for a film of tellurium nanowires produced via electroless deposition from 5 mM TeI_4 in 12CE-I DES at 80°C.

X-Ray Diffraction Spectroscopy (XRD)

Crystallographic analysis of the tellurium films was done for the electroless deposition of 5 mM $TeCl_4$ from 12CE-Cl DES. Deposition was done in a thermostatically controlled bath at 80°C for 4 hours. Sharp peaks indicate a high degree of crystallinity.

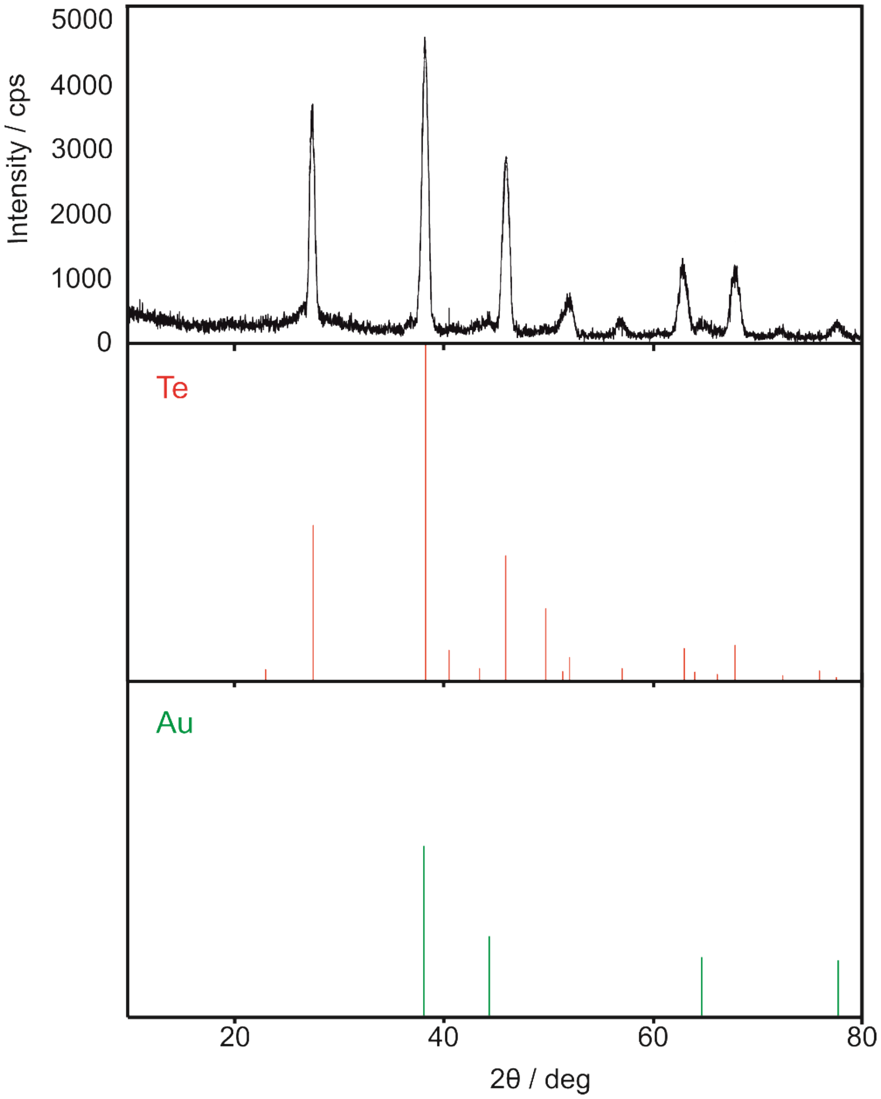

Figure S4. Black) XRD pattern of a representative Te nanowire film produced by a 4 hour electroless deposition in 5 mM TeCl4, 12CE-Cl at 80°C. Red) Pure Te phase (ICDD 00-085-0554). Green) Pure Au phase to indicate peaks from the substrate (ICDD 00-004-0784).
